# OH^−^ absorption and holographic storage properties of Sc(0, 1, 2, 3):Ru:Fe:LiNbO_3_ crystals

**DOI:** 10.1039/c7ra12606a

**Published:** 2018-01-30

**Authors:** Li Dai, Luping Wang, Chunrui Liu, Xianbo Han, Zhehua Yan, Yuheng Xu

**Affiliations:** College of Science, Harbin University of Science and Technology Harbin 150080 China daili198108@126.com +86 451 86390731; School of Materials Science and Engineering, Harbin University of Science and Technology Harbin 150040 China; Department of the Applied Chemistry, Harbin Institute of Technology Harbin 150001 China

## Abstract

A series of Sc:Ru:Fe:LiNbO_3_ crystals with various levels of Sc_2_O_3_(0, 1, 2, and 3 mol%) doping were grown from congruent melts in air by using the Czochralski technique. The defect structures and photorefractive properties of the Sc:Ru:Fe:LiNbO_3_ crystals were investigated by acquiring infrared spectra of the crystals and performing two-wavelength nonvolatile experiments, respectively. Our results showed the holographic storage properties of Ru:Fe:LiNbO_3_ crystals to be enhanced by doping them with a high concentration of Sc_2_O_3_, and indicated Sc:Ru:Fe:LiNbO_3_ crystals to constitute a promising medium for holographic storage.

## Abstract 2

A series of Sc:Ru:Fe:LiNbO_3_ crystals with various levels of Sc_2_O_3_(0, 1, 2, and 3 mol%) doping were grown from congruent melts in air by using the Czochralski technique.

## Graphical abstract

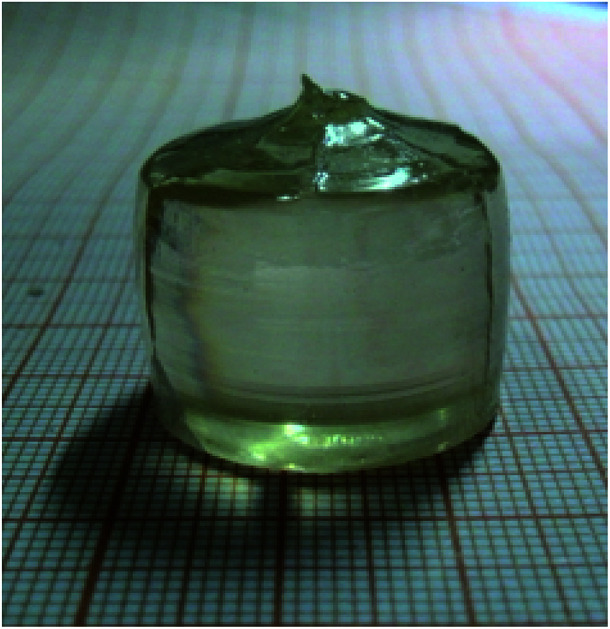

## 1. Introduction

With excellent nonlinear optical, electro-optical, acoustic-optical, ferroelectric and photorefractive properties, LiNbO_3_(LN) crystals have become very promising materials.^1,2^ In the past few decades, due to its high storage capacity, fast parallel processing and content addressability, the LiNbO_3_ crystal has garnered great interest,^3^ and has been successfully applied in integrated electro-optical devices and holographic memory devices.^4^ However, the volatility of stored information is a major obstacle in the practical application of LiNbO_3_ crystals. In order to solve this problem, photorefractive ions such as those of Fe,^5^ Ce,^6^ Cu,^7^ Ru,^8^ Mn, Ti,^9^ Er^10–14^ are introduced into the crystal to enhance the photorefractive effect, and it was found that a nonvolatile readout can be realized in some doubly doped LiNbO_3_.^15,16^

Based on this development, a two-centered recording model was proposed in the doubly doped Fe:Mn:LiNbO_3_ crystal by Buse *et al.* in 1998,^17^ and holographic recording and nondestructive readout were implemented in Fe:Mn:LiNbO_3_. The key point of the technique is that the doped ions can provide both relatively shallow and deep centers in the crystals. Many new doubly doped crystals, such as Cu:Ce:LiNbO_3_, Mn:Ce:LiNbO_3_ and Fe:Cu:LiNbO_3_ crystals, have since been reported to have such properties.^18,19^ The Ru:Fe:LiNbO_3_ crystal was recently found to be another excellent medium for holographic storage. Fe and Ru are both transition mental ions and are located at similar positions of the periodic table; and in the Ru:Fe:LiNbO_3_ crystal, Ru is used as a deep center and Fe as a shallow center.^20^ Generally speaking, the higher the concentration of the doping ion, the stronger the photorefractive effect; therefore, a lower response time, higher sensitivity, higher diffraction efficiency and other excellent parameters can be achieved by using a higher doping concentration. But it is not easy to grow large and high-quality Ru:Fe:LiNbO_3_ crystals, because of the low solubility of Fe and Ru in the LiNbO_3_ crystal. Comparatively speaking, doping ions resistant to optical damage is a more feasible method: it not only can eliminate the intrinsic defects caused by the Li composition deficiency but also can improve optical resistance ability of the crystal.

The choice of doping ions is critical for the characteristics and applications of LN crystals. In this work, the Sc^3+^ (ref. 21–24) ion was chosen as the doping ion. The ionic radius of Sc^3+^ is similar to that of Li^+^ but larger than that of Nb^5+^, and the Sc^3+^-doped LN has been used in integrated optics such as titanium-diffused optical LN waveguides doped with Sc^3+^. The LN crystal is susceptible to photorefractive damage under laser irradiation. To suppress photorefractive damage, the LN crystal must be doped with more than 4.6 mol% Mg,^25^ more than 6.2 mol% Zn,^26^ more than 3 mol% In,^27^ or more than 2 mol% Sc.^28^ We chose Sc^3+^ ion as the doping ion since it was effective at a concentration lower than were any of the other ions.

Based on the above considerations, a series of Sc:Ru:Fe:LiNbO_3_ crystals with various concentrations of Sc_2_O_3_ were grown by using the Czochralski method. The defect structures of Sc:Ru:Fe:LiNbO_3_ crystals were investigated by acquiring their infrared spectra, and the holographic storage properties of Sc:Ru:Fe:LiNbO_3_ were investigated by taking two-wavelength nonvolatile measurements.

## 2. Crystal growth

**Fig. 1:**
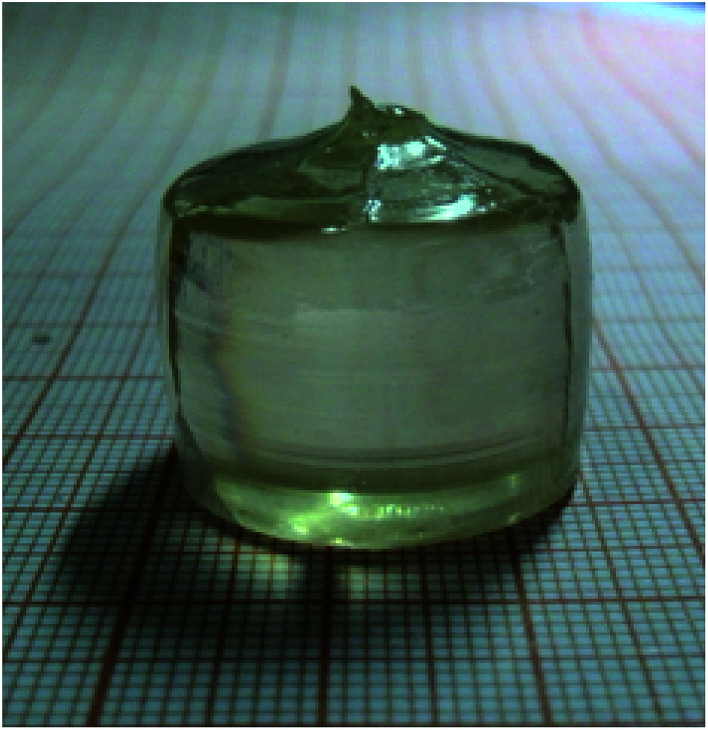
A sample ScRuFe-3 crystal.

In our experiment, the Sc(0, 1, 2, and 3 mol%):Ru:Fe:LiNbO_3_ crystals with 0.3 mol% Fe_2_O_3_ and 0.2 mol% RuO_2_ were grown from congruent melts in air by using the Czochralski technique. The raw materials used in crystal growth were Nb_2_O_5_, LiCO_3_, Sc_2_O_3_, RuO_2_ and Fe_2_O_3_. The purity of raw material is critical for optical quality, so the purity levels of all raw materials were at least 99.99%. All raw materials including Nb_2_O_5_, LiCO_3_, Sc_2_O_3_, RuO_2_ and Fe_2_O_3_ were mixed for 24 hours in order to obtain uniform materials. Then the materials were placed into a platinum crucible heated up to 750 °C for 2 hours to remove CO_2_ and then heated up further to 1150 °C for 2 hours to form a polycrystalline material as a result of a solid-state reaction. The growth condition was selected as follows: the temperature gradient was 2.5 °C mm^−1^, the polling rate and rotation rate were controlled to be in the range 0.8 to 1.5 mm h^−1^ and 17–26 rpm, respectively. After growth, the crystals were cooled to room temperature at a rate of 65 °C h^−1^. In order to prevent spontaneous polarization of the crystals, all of the crystals needed to be polarized artificially in a medium frequency furnace for 8 h, in which the temperature was 1100 °C, the temperature gradient was almost equal to zero, and the current density was 5 mA cm^−2^. Several 8 mm × 10 mm × 2 mm (*x* × *y* × *z*) wafers were obtained by cutting from the middle of the Sc:Ru:Fe:LiNbO_3_ crystals along the *y*-axis. The samples with different Sc^3+^ ion concentrations were labeled as ScRuFe-0, 1, 2 and 3. A photograph of one of the ScRuFe-3 crystals is shown in Fig. 1.

## 3. Measurements

### 3.1 Infrared absorption spectra of Sc:Ru:Fe:LiNbO_3_ crystals

The water in the raw materials and growth atmosphere were expected to cause H^+^ ions to enter the crystal lattice and form O–H–O during the crystal growth. The frequency and energy of infrared light can only make molecules vibrate and their rotation levels change. Since the vibration of O–H is very sensitive to its environment, including surrounding ions, we expected the defects and structure of our crystals to be amenable to analysis using infrared spectroscopy.^29^

**Fig. 2:**
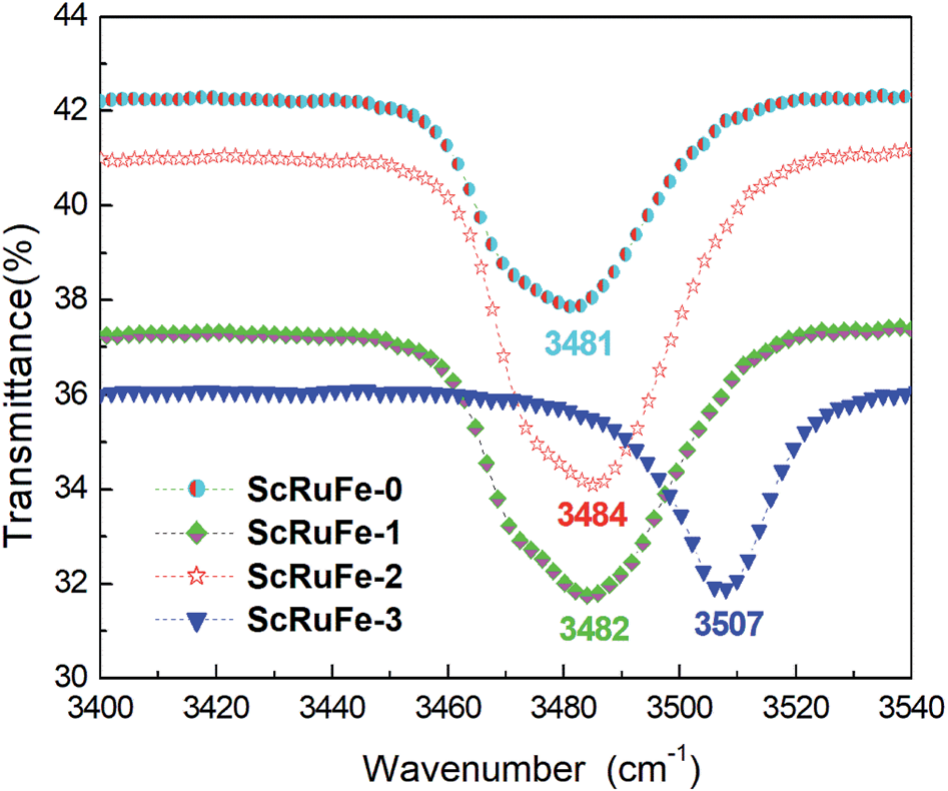
Infrared transmittance spectra of Sc:Ru:Fe:LiNbO_3_ crystals.

The infrared absorption spectra of the crystals were each acquired in the wavenumber range 3400 cm^−1^ to 3540 cm^−1^ at room temperature. As shown in Fig. 2, the OH^−^ absorption bands of samples ScRuFe-0, 1 and 2 were all located at about 3482 cm^−1^, while the OH^−^ band of sample ScRuFe-3 was located at 3507 cm^−1^. The shape of an OH^−^ absorption band is related to the crystal composition and ions surrounding the OH^−^. The OH^−^ absorption band of LiNbO_3_ is also located at 3482 cm^−1^.^30^

The mechanism underlying the shift of the absorption peak can be described as follows. The [Li]/[Nb] ratio in congruent LiNbO_3_ crystals has been previously indicated to be about 0.946, and to have caused the generation of intrinsic defects. During the growth of such crystals, a lattice position missing the Li^+^ ion would form a Li vacancy V_Li_^−^, which would be filled by a Nb^3−^ ion to form an anti site Nb Nb_Li_^4+^. The concentration threshold of anti-photorefraction can be defined as that when the doped ions enter the normal Nb position. In our experiment, the concentrations of Fe^3+^ and Ru^4+^ ions were fixed, so we just took the Sc^3+^ ion into account; as indicated above, the concentration threshold of Sc^3+^ ions has been reported to be 2 mol%. The concentrations of Sc_2_O_3_ added into the growth melts of samples ScRuFe-0, 1, 2 and 3 were measured using ICP-AES spectrometry to be 0 mol%, 0.78 mol%, 1.44 mol% and 2.07 mol% respectively. For the Sc:Ru:Fe:LiNbO_3_ crystals with a dopant concentration below its threshold value, the doping Sc, Ru and Fe ions, according to the proposed mechanism, replaced Nb_Li_^4+^ and V_Li_^−^ to form the defects Sc_Li_^2+^, Ru_Li_^3+^ and Fe_Li_^2+^, respectively. Due to the repulsion between these ions and H^+^, the H^+^ ions were still attracted by V_Li_^−^, which caused the OH^−^ absorption bands of samples ScRuFe-0, 1, 2 to be quite similar. According to the proposed mechanism, for the concentration of Sc^3+^ ions exceeding the threshold, Sc^3+^ occupied Nb sites and formed the defect Sc_Nb_^2−^. Sc_Nb_^2−^ attracts H^+^ ions more strongly than does V_Li_^−^, so the H^+^ ions gathered near Sc_Nb_^2−^ according to our proposal. The shift of the absorption band of sample ScRuFe-3 to 3507 cm^−1^ was attributed to the above mechanism. Note also that the OH^−^ absorption band of sample ScRuFe-3 was sharper than those of the others, and this observation was attributed to the formation of more Sc_Nb_^2−^ defects resulting from the higher concentration of Sc^3+^ in this sample.

### 3.2 Two-wavelength nonvolatile measurements

**Fig. 3:**
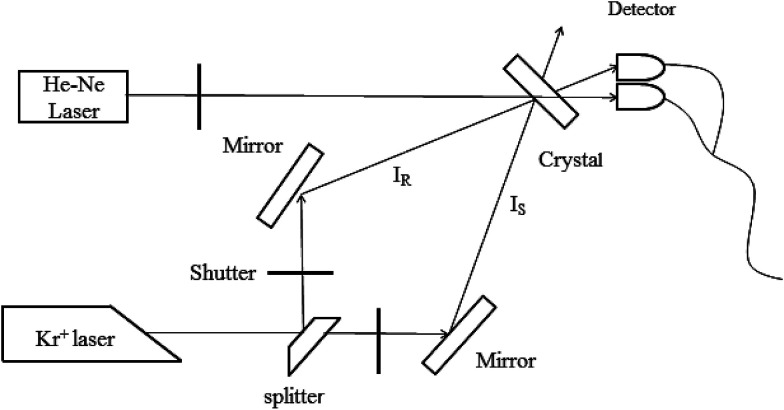
Two-wavelength nonvolatile experiment setup.

Two-wavelength nonvolatile measurements were taken to study the holographic storage properties of the Sc:Ru:Fe:LiNbO_3_ crystals. The experimental setup is shown in Fig. 3. A Kr^+^ laser with *λ* = 476 nm and an He–Ne laser with *λ* = 633 nm were used as the recording and readout beams, respectively. By using a continuously adjustable beam splitter, the recording beam was split into two beams, *I*_S_ and *I*_R_, of equal 120 mW cm^−2^ intensity. The two beams were then polarized in the incidence plane, and then directed to the crystal at the corresponding Bragg angle of 16°. The two beams intersected symmetrically inside the crystal and made the grating vector along the *c*-axis. During the recording process, the two beams were first directed onto the crystal at the same time, and then the beam *I*_S_ was blocked from time to time by a shutter, and the diffraction efficiency of another beam *I*_R_ was detected for a short duration of 10 s in order to eliminate the impact of erasure. After the grating store was saturated, *I*_S_ and *I*_R_ were both blocked only by the He–Ne beam directed onto the sample, and the intensities of transmitted *I*_t_ and diffracted *I*_d_ were determined. This process was the nonvolatile readout. By carrying out these experiments, the holographic storage properties of the samples were determined.

Next we discuss the holographic storage properties of the Sc:Ru:Fe:LiNbO_3_ crystals.

The diffraction efficiency *η* was defined as, the diffraction efficiency *η* was defined as

1 *η* = *I*_d_/*I*_t_ × 100%

where *I*_t_ is the transmitted light intensity and *I*_d_ the diffracted light intensity of the readout beam.2
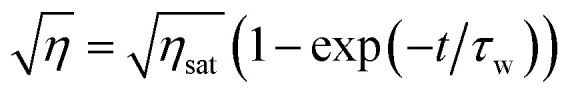


The recording and erasure time constants were described by using eqn (2) and (3).3
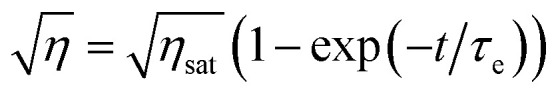


In these equations, *τ*_w_ and *τ*_e_ are the recording and erasure time constants respectively. Also, *η*_sat_ is the saturation diffraction efficiency during recording, and it was fixed by the function4
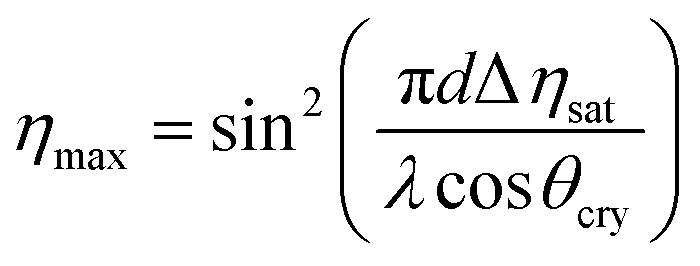
where *η*_max_ is the maximum value of diffraction efficiency, *d* is the thickness of the samples, *λ* is the wavelength of the recording beam, and *θ*_cry_ is the refraction angle of incidence light within the crystal.

Sensitivity (*S*) and its dynamic range (*M*/#) were calculated using eqn (5) and (6).^31,32^5
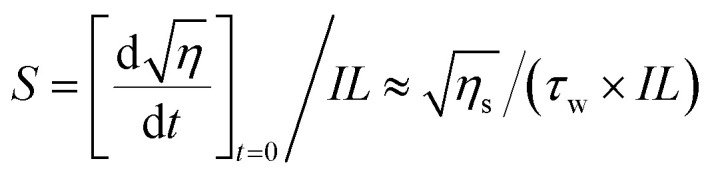
6
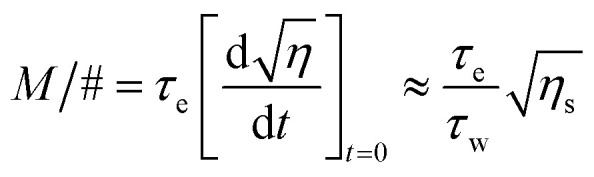
In these equations, *I* is the total optical intensity, and *L* is the crystal plate thickness.

**Fig. 4:**
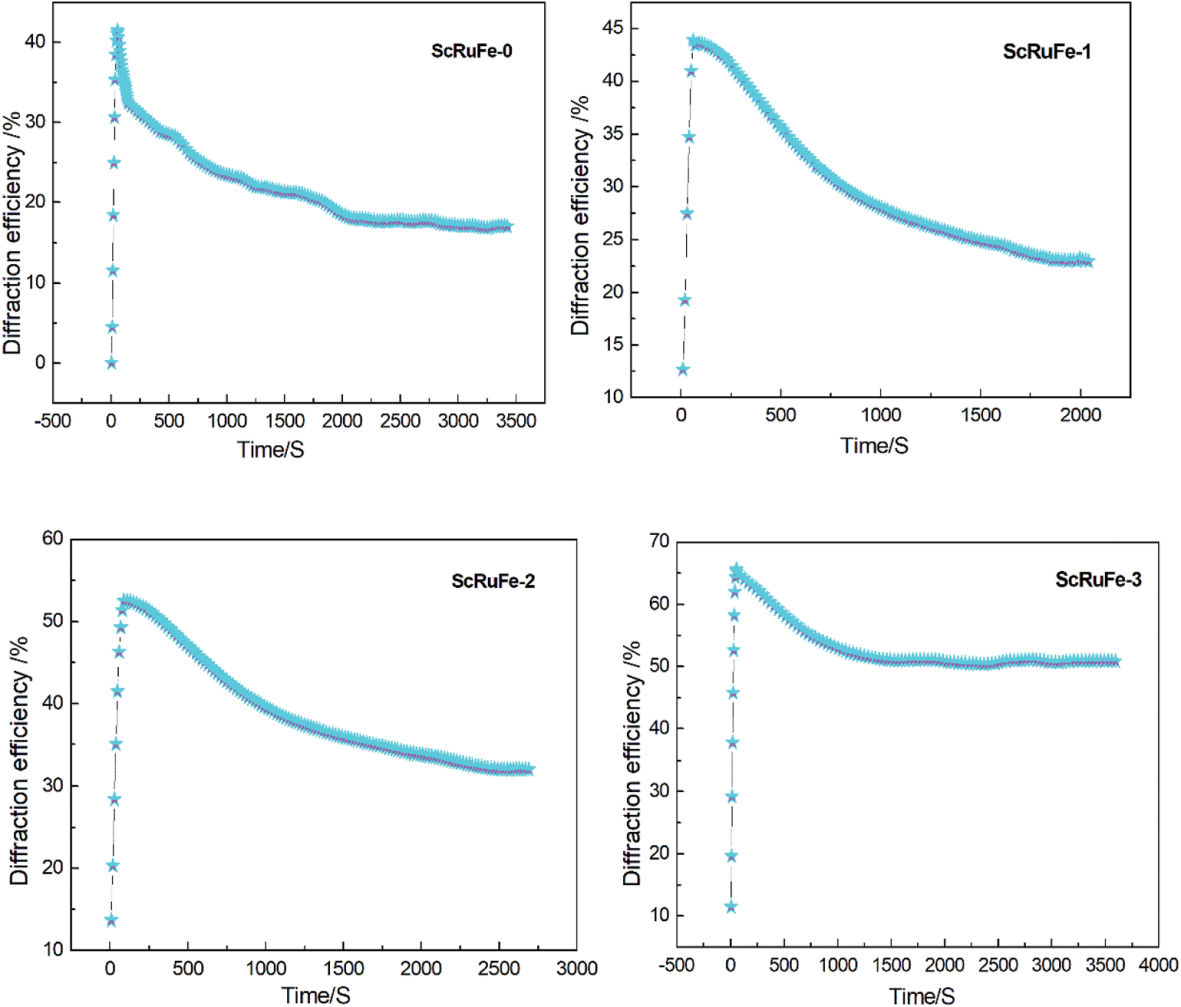
Time dependence of diffraction efficiency during dual-wavelength nonvolatile holographic recording of the samples.

The dual-wavelength nonvolatile holographic recording-readout curves are shown in Fig. 4.

The holographic storage parameters of the Sc:Ru:Fe:LiNbO_3_ crystals measured by performing the dual-wavelength experiment are listed in Table 1. The results showed that the holographic storage parameters improved as the Sc_2_O_3_ doping concentration was increased. Compared to the values of ScRuFe-0, *τ*_w_ of ScRuFe-3 decreased by a factor of 3.0, *η*_s_ increased by a factor of 1.6, *S* increased by a factor of 4, and *M*/# increased by a factor of 7.9. The *η*_s_, *S*, and *M*/# values of sample ScRuFe-3 were in fact higher than the corresponding values of the other tested samples.

**Table 1:** Experimentally determined holographic storage properties of the Sc:Ru:Fe:LiNbO_3_ crystals

Sample	*τ*_w_ (s)	*τ*_e_ (s)	*η*_s_ (%)	*S* (cm J^−1^)	*M*/#	Δ*n*_s_ (× 10^−5^)	*σ*_ph_ (× 10^−12^ cm Ω^−1^ W^−1^)
ScRuFe-0	389.2	937.2	41.2	0.04	1.55	4.17	0.64
ScRuFe-1	275.1	1105.7	44.5	0.06	2.68	4.33	0.90
ScRuFe-2	210.5	1461.5	53.4	0.09	5.07	4.75	1.18
ScRuFe-3	128.9	1937.8	65.6	0.16	12.18	5.26	1.92

The holographic storage properties of the Sc:Ru:Fe:LiNbO_3_ crystal are known to depend on the photorefractive sensitive center. In this crystal, there are two photorefractive ions, Ru^4+^ and Fe^3+^, which can form deep and shallow trap centers, respectively. The Sc^3+^ ion has a stable single valence state, so it cannot form a photorefractive center. First we discuss the carrier transport model in Sc:Ru:Fe:LiNbO_3_ crystals. Here, Ru and Fe both have two different valences, which can form a donor level and acceptor level. Ru^3+^ and Fe^2+^ ions can bind many donor level electrons, while Ru^4+^ and Fe^3+^ can bind almost no accepter level electrons. When the incidence light was directed onto the crystal, the electrons in the donor level (Ru^3+^ and Fe^2+^) were excited to the conduction band, and after drift and diffusion, they were absorbed by accepter levels (Ru^4+^ and Fe^3+^). The process can be described as follows:^33^
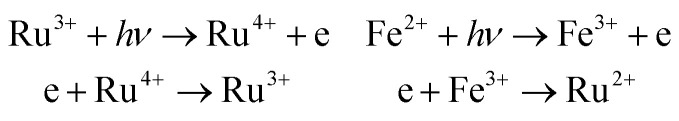


The Sc^3+^ ion enter into the lattice of Ru:Fe:LiNbO_3_ crystal has no direct influence on the formation of the grating. The photorefractive ions Fe^2+^/Fe^3+^ and Ru^3+^/Ru^4+^ play a dominate role in photo-excited carrier transport processes. Doped Sc^3+^ ions influenced the ion arrangement and defects in the Sc:Ru:Fe:LiNbO_3_ crystals, and a detailed mechanism for this influence was derived. According to this proposed mechanism, Sc^3+^ ions at doping concentrations below its threshold concentration replaced the Nb_Li_^4+^ defects, while Sc^3+^ ions at concentrations exceeding its threshold concentration replaced the normal Li position. Due to the polarization ability of the Sc^3+^ ion being stronger than that of the Li^+^ ion, the ability of Sc^3+^ ions to capture electrons was also better than that for Li^+^ ions. In the process, the trap density of the electron acceptor increased and the saturation diffraction efficiency *η*_sat_ was also improved. The equation mentioned above indicated the dynamic range *M*/# to be approximately proportional to the saturation diffraction efficiency *η*_sat_, so the dynamic range *M*/# increased with increasing concentration of the doped Sc^3+^ ions. For the LiNbO_3_ crystal, the photoconductivity *σ*_ph_ has been shown to be related to the electron traps and to be inversely proportional to the Nb_Li_^4+^ concentration. Increasing the concentration of the doped Sc^3+^ ions led to a decrease in the concentration of Nb_Li_^4+^, which in turn led to the increase in the photoconductivity *σ*_ph_ and decrease in the response time. Sensitivity *S* is the comprehensive measure of saturation diffraction efficiency *η*_sat_ and photoconductivity *σ*_ph_. As the results showed, increasing the concentration of Sc^3+^ doped into the Ru:Fe:LiNbO_3_ crystals coincided with a decrease in writing time *τ*_w_ and increases in the dynamic range *M*/#, saturation diffraction efficiency *η*_sat_, photoconductivity *σ*_ph_ and sensitivity *S*.

## 4. Conclusions

Sc:Ru:Fe:LiNbO_3_ crystals with various concentrations of Sc^3+^ were grown by using the Czochralski method. The OH^−^ absorption experiment results showed the absorption bands of samples ScRuFe-0, 1, 2 to all be located at a similar wavenumber, of about 3484 cm^−1^, when the doping Sc^3+^ ion concentration was below its threshold concentration. Once the Sc^3+^ concentration exceeded its threshold value, *i.e.*, for ScRuFe-3, the absorption band shifted significantly, to 3507 cm^−1^. The two-wavelength nonvolatile experiment results demonstrated that the holographic storage properties improved with increasing Sc^3+^ concentration. Compared to the other samples, ScRuFe-3, *i.e.*, that with the highest Sc^3+^ doping concentration, showed the shortest response time, and the highest dynamic range *M*/#, saturation diffraction efficiency *η*_sat_, and sensitivity levels, which are key parameters of volume holographic date storage. These results indicated the Sc:Ru:Fe:LiNbO_3_ crystals to be promising materials for nonvolatile holographic storage.

## Conflicts of interest

There are no conflicts to declare.

## Supplementary Material
